# Supplementary material for: Printing via Laser-Induced Forward Transfer and the Future of Digital Manufacturing
Source: Materials (Basel). 2023 Jan 11;16(2):698. doi: 10.3390/ma16020698 (PMC9865182; doi:10.3390/ma16020698)
Supplement: Supplementary file 1 [file materials-16-00698-s001.zip › materials-2070810-supplementary.pdf]

Printing via Laser-Induced Forward Transfer and the Future of Digital Manufacturing

Camilo Florian [1,2,*] and Pere Serra [3]

[1] Princeton Institute for the Research and Technology of Materials (PRISM), Princeton University, 70 Prospect Av, Princeton, NJ 08540, USA
[2] Instituto de Óptica Daza de Valdés, Consejo Superior de Investigaciones Científicas (IO-CSIC), Calle Serrano 122, 28006 Madrid, Spain
[3] Departament de Fisica Aplicada, Universitat de Barcelona, Martí i Franqués 1, 08028 Barcelona, Spain
* Correspondence: camilo.florian@csic.es

Supplementary Material

1. Results of patent and patent application lists sorted using the keywords "laser induced forward transfer" into two public patent office data bases: the European Patent Office (EPO) and the United States Patent and Trademark Office (USPTO). On this premise, we select patents publications that are publicly accessible and published with a patent publication number, regardless of the acceptance or licensing status. In the following data we therefore jointly present patents with patent applications. We find 321 patents reported on the EPO and 303 on USPTO, that, regardless of the publication country, are reported in these databases.

Patent list from EPO

#	Publication number	Title	Earliest publication
1	US2022348859A1	LITHOGRAPHIC MASKING FOR SPATIALLY LOCALIZED BIOCHEMICAL STIMULUS DELIVERY	2022-11-03
2	CN115181962A	A LASER-INDUCED HIGH-VISCOSITY PASTE MULTIPLE IMPRINTING METHOD	2022-10-14
3	JP2022151818A	DISPLAY DEVICE MANUFACTURING METHOD	2022-10-07
4	WO2022202945A1	METHOD FOR MANUFACTURING DISPLAY DEVICE	2022-09-29
5	CN115094374A	MATERIALS AND METHODS FOR MAKING PATTERNED METAL OXIDE THIN FILMS	2022-09-23
6	US2022296424A1	SYSTEM AND METHOD FOR PERSONALIZED IMPLANTABLE SCAFFOLDS FOR WOUND HEALING	2022-09-22
7	CN115041836A	A LASER-INDUCED TRANSFER METHOD FOR MAGNETIC DRIVE UNIT OF A MAGNETIC DRIVE ORIGAMI SOFT ROBOT	2022-09-13
8	US2022281253A1	ADDITIVE OPTO-THERMOMECHANICAL NANOPRINTING AND NANOREPAIRING UNDER AMBIENT CONDITIONS	2022-09-08
9	CN114944442A	A LASER PROJECTION PROXIMITY TYPE MICROLED MASS TRANSFER TRANSPOSITION, METHOD AND SYSTEM	2022-08-26
10	US2022266382A1	LASER-SEEDING FOR ELECTRO-CONDUCTIVE PLATING	2022-08-25
11	US2022260063A1	ELECTROSPRAY EMISSION APPARATUS	2022-08-18
12	WO2022167516A1	A METHOD TO PREPARE POCKETS OF ENCAPSULATED MATERIAL COMPRISING A CORE SURROUNDED BY AN ENCAPSULATION	2022-08-11
13	CN114864474A	A LASER PROJECTION PROXIMITY MASS TRANSFER DEVICE, METHOD AND EQUIPMENT	2022-08-05
14	US2022248540A1	HIGH-RESOLUTION SOLDERING	2022-08-04
15	JP7111916B1	RETRANSFER METHOD AND LIFT METHOD	2022-08-02
16	CN114799225A	PULSE LASER DRIVING METAL MICRODROPLET PRINTING SYSTEM AND ADJUSTING METHOD	2022-07-29

**17**	US2022227116A1	FOUNTAIN SOLUTION IMAGING USING DRY TONER ELECTROPHOTOGRAPHY	2022-07-21
**18**	US11393773B1	STRESS ISOLATING INTERPOSER AND SENSOR PACKAGE AND METHOD OF MANUFACTURING THE SAME	2022-07-19
**19**	WO2022144608A1	LIFT PRINTING OF FINE METAL LINES	2022-07-07
**20**	WO2022147143A1	CONFIGURABLE LEADED PACKAGE	2022-07-07
**21**	US2022208701A1	PRINTED PACKAGE AND METHOD OF MAKING THE SAME	2022-06-30
**22**	US2022194111A1	METHOD AND A DEVICE FOR ASSEMBLY OF A NANOMATERIAL STRUCTURE	2022-06-21
**23**	KR20220081748A	OPTICAL ELEMENT ARRAY STRUCTURE FOR BIRD COLLISION PREVENTION AND MANUFACTURING METHOD THEREOF	2022-06-09
**24**	JP2022086606A	FLYING OBJECT GENERATION DEVICE, IMAGE FORMATION DEVICE, SOLID MOLDING MANUFACTURING APPARATUS, FLYING OBJECT GENERATION METHOD, AND SUBSTRATE FOR LIGHT ABSORPTION MATERIAL FLYING	2022-06-09
**25**	JP2022086831A	FLYING OBJECT GENERATION METHOD, FLYING OBJECT GENERATION DEVICE, IMAGE FORMATION APPARATUS AND MANUFACTURING DEVICE OF THREE-DIMENSIONALLY MOLDED ARTICLE	2022-06-02
**26**	WO2022106914A1	REPAIR OF SOLDER BUMPS	2022-05-27
**27**	WO2022104099A1	TRANSDUCERS, THEIR METHODS OF MANUFACTURE AND USES	2022-05-19
**28**	WO2022063393A1	METHOD AND 3D PRINTING METHOD FOR LAYER-BY-LAYER FABRICATION OF OBJECTS USING LAYER TRANSFER PRINTING	2022-03-31
**29**	US2022090022A1	GENERATING INDUCED PLURIPOTENT STEM CELLS	2022-03-24
**30**	CN114178547A	LASER-INDUCED TRANSFER MICRO ELECTRONIC COMPONENT PRINTING METHOD BASED ON NON-NEWTONIAN FLUID CHARACTERISTICS	2022-03-15
**31**	US2022040377A1	LASER ABLATION/REMOVAL AND LASER INDUCED FORWARD TRANSFER OF BIOLOGICAL MATERIAL	2022-02-10
**32**	JP2022026065A	FLYING OBJECT GENERATION DEVICE, IMAGE FORMATION DEVICE, MANUFACTURING METHOD OF THREE-DIMENSIONAL SHAPED ARTICLE, AND FLYING OBJECT GENERATION METHOD	2022-02-10
**33**	WO2022003431A1	LASER PRINTING OF SOLDER PASTES	2022-01-06
**34**	EP3933902A1	A METHOD FOR POSITIONING COMPONENTS ON A SUBSTRATE	2022-01-05
**35**	EP3928993A1	DEVICE FOR APPLYING ENERGY TO A SUBSTRATE	2021-12-28
**36**	CN215151845U	SOLUTION BATH NEAR-FIELD CELL 3D PRINTING FORMING DEVICE	2021-12-14
**37**	JP2021187143A	LIGHT IRRADIATION METHOD, APPARATUS FOR ATTACHING LIGHT ABSORBING MATERIAL, FLYING BODY GENERATING METHOD AND APPARATUS, IMAGE FORMING METHOD, AND THREE-DIMENSIONAL OBJECT PRODUCING METHOD	2021-12-13
**38**	WO2021245467A1	HIGH-RESOLUTION SOLDERING	2021-12-09
**39**	US2021379581A1	MAGNETIC PCR ASSAY AND USES THEREOF	2021-12-09
**40**	CN113733547A	LIGHT IRRADIATION METHOD, LIGHT ABSORBING MATERIAL ATTACHMENT DEVICE, AND RELATED METHODS AND DEVICES	2021-12-02
**41**	WO2021239765A1	TOPOGRAPHY-BASED DEPOSITION HEIGHT ADJUSTMENT	2021-12-01
**42**	CN113547736A	MULTI-MATERIAL LASER-INDUCED TRANSFER 3D PRINTING METHOD AND DEVICE	2021-10-26
**43**	KR102373098B1<br>KR20210127522A	LIGHT EMITTING DEVICE	2021-10-22
**44**	KR102360514B1<br>KR20210127523A	LIGHT EMITTING DEVICE	2021-10-22
**45**	JP2021151772A	FLYING BODY GENERATION METHOD, FLYING BODY GENERATION APPARATUS AND IMAGE FORMATION METHOD	2021-09-30
**46**	JP2021151666A	SCAN TYPE OPTICAL REDUCTION SYSTEM AND LASER PROCESSING DEVICE USING THE SAME	2021-09-30
**47**	WO2021191897A1	COLLAGEN-BASED FORMULATIONS USABLE AS SOFT TISSUE FILLERS AND/OR IMPLANTS	2021-09-30
**48**	KR102373099B1<br>KR20210112880A	LIGHT EMITTING DEVICE AND METHOD OF MANUFACTURING THE SAME	2021-09-15
**49**	KR102357759B1<br>KR20210112882A	LIGHT EMITTING DEVICE AND METHOD OF MANUFACTURING THE SAME	2021-09-15
**50**	KR20210112878A	LIGHT EMITTING DEVICE AND METHOD OF MANUFACTURING THE SAME	2021-09-15
**51**	GB2591498A<br>GB2591498B	A METHOD OF CONNECTING CIRCUIT ELEMENTS.	2021-08-04
**52**	KR102378115B1<br>KR20210089555A	METHOD OF TRANSFERRING LIGHT EMITTING DEVICE CHIP	2021-07-16
**53**	KR102325792B1<br>KR20210089557A	LIGHT EMITTING DEVICE AND METHOD OF MANUFACTURING THE SAME	2021-07-16

**54**	KR102315912B1<br>KR20210089558A	METHOD OF MANUFACTURING LIGHT EMITTING DEVICE	2021-07-16
**55**	KR102315911B1<br>KR20210089556A	LIGHT EMITTING DEVICE AND METHOD OF MANUFACTURING THE SAME	2021-07-16
**56**	CN113085185A	FEMTOSECOND LASER-INDUCED LIQUID FILM FORWARD TRANSFER METHOD BASED ON TEMPORAL SHAPING	2021-07-09
**57**	US2021205813A1	CONTACTLESS LIQUID LOADING TO MICROFLUIDIC DEVICES	2021-07-08
**58**	KR102437637B1<br>KR20210084120A	METHOD OF MANUFACTURING LIGHT EMITTING DEVICE	2021-07-07
**59**	KR102403425B1<br>KR20210084124A	METHOD OF MANUFACTURING MICRO LED DISPLAY	2021-07-07
**60**	KR102301877B1<br>KR20210084119A	LIGHT EMITTING DEVICE	2021-07-07
**61**	KR20210084122A	METHOD OF MANUFACTURING LIGHT EMITTING DEVICE	2021-07-07
**62**	CN113021874A	SINGLE CELL PRINTING METHOD BASED ON ANNULAR LASER SPOT INDUCED TRANSFER	2021-06-25
**63**	KR102397517B1<br>KR20210070834A	LIGHT EMITTING DEVICE	2021-06-15
**64**	KR102325791B1<br>KR20210070835A	LIGHT EMITTING DEVICE	2021-06-15
**65**	KR102301879B1<br>KR20210070833A	METHOD OF MANUFACTURING A LIGHT EMITTING DEVICE	2021-06-15
**66**	CN114762133A	METHOD FOR MANUFACTURING SEMICONDUCTOR LIGHT-EMITTING ELEMENT	2021-06-10
**67**	WO2022160506A1	DROPLET THREE-DIMENSIONAL PRINTING SYSTEM AND METHOD DRIVEN BY PULSED LASER	2021-06-08
**68**	US2021168943A1	METHOD FOR MANUFACTURING A CONDUCTOR STRUCTURAL ELEMENT AND CONDUCTOR STRUCTURAL ELEMENT	2021-06-03
**69**	US2021157238A1	METHODS AND SYSTEMS FOR PRODUCING THREE-DIMENSIONAL ELECTRONIC PRODUCTS	2021-05-27
**70**	US2021155023A1	ARCHITECTED STAMPS FOR LIQUID TRANSFER PRINTING	2021-05-27
**71**	US2021139321A1	LASER-ASSISTED MATERIAL PHASE-CHANGE AND EXPULSION MICRO-MACHINING PROCESS	2021-05-13
**72**	CN112768572A<br>CN112768572B	MINIATURE LED MASS TRANSFER METHOD AND DEVICE BASED ON HIGH-SPEED SCANNING LASER TRANSFER PRINTING	2021-05-07
**73**	WO2021090089A1	SYSTEMS FOR MATERIAL DEPOSITION	2021-05-06
**74**	US2022293577A1	DISPLAY DEVICE AND DISPLAY UNIT	2021-04-08
**75**	JP2021053860A	THREE-DIMENSIONAL MODELING APPARATUS, CARRIER, AND THREE-DIMENSIONAL MODELING METHOD	2021-04-08
**76**	CN212793450U	SOLDER PASTE LASER-INDUCED FORWARD TRANSFER EQUIPMENT	2021-03-26
**77**	US2021090945A1	SYSTEM AND METHOD FOR INTERCONNECTION	2021-03-25
**78**	CN112477110A	SOLUTION BATH NEAR-FIELD CELL 3D PRINTING FORMING DEVICE AND FORMING METHOD THEREOF	2021-03-12
**79**	CN114270508A	METHOD AND SYSTEM FOR STRETCHING RECEPTOR SUBSTRATE TO ADJUST ARRANGEMENT OF COMPONENTS	2021-02-25
**80**	US11214015B2<br>US2021053297A1	METHODS AND SYSTEMS FOR CONTROLLING TEMPERATURE ACROSS A REGION DEFINED BY USING THERMALLY CONDUCTIVE ELEMENTS	2021-02-25
**81**	CN112382676A<br>CN112382676B	SOLAR CELL GRID LINE LASER-INDUCED PRINTING METHOD BASED ON SILICON WAFER DOUBLE-GROOVE STRUCTURE	2021-02-19
**82**	US2021032739A1	DYNAMIC RELEASE MIRROR STRUCTURE FOR LASER-INDUCED FORWARD TRANSFER	2021-02-04
**83**	EP3771547A1	METHOD FOR 3D PRINTING OF VASCULARIZED TISSUES AND ORGANS	2021-02-03
**84**	CN112140530A<br>CN112140530B	MULTI-MODULE SERIAL BIOLOGICAL 3D PRINTING DEVICE	2020-12-29
**85**	WO2020254782A1	METHOD OF MANUFACTURING A MICROFLUIDIC ARRANGEMENT, METHOD OF OPERATING A MICROFLUIDIC ARRANGEMENT, APPARATUS FOR MANUFACTURING A MICROFLUIDIC ARRANGEMENT	2020-12-24
**86**	WO2021005326A1	CONTACT LENSES WITH MICROCHANNELS	2020-12-10
**87**	WO2020225810A1	LIFT PRINTING USING THIN DONOR FOILS	2020-11-12
**88**	WO2020225777A1	SYSTEM AND METHOD FOR ORGANOID CULTURE	2020-11-12
**89**	CN111910154A<br>CN111910154B	DYNAMIC FREQUENCY SELECTIVE SURFACE STRUCTURE AND PREPARATION METHOD THEREOF	2020-11-10
**90**	WO2020222090A1	METHOD TO ELECTRICALLY CONNECT CHIP WITH TOP CONNECTORS USING 3D PRINTING	2020-11-05
**91**	WO2020212802A1	ANTERIOR LAMELLAR KERATOPLASTY	2020-10-22
**92**	WO2020212199A1	DEVICES AND METHODS FOR LASER SURGERY OF AN EYE, ESPECIALLY FOR KERATOPLASTY	2020-10-22
**93**	US10808794B1	TOPOLOGICAL DAMPING MATERIALS AND METHODS THEREOF	2020-10-20

**94**	KR20210143883A	ADDITIVE MANUFACTURING ELECTRONIC (AME) CIRCUITS WITH SIDE-MOUNTED COMPONENTS	2020-10-08
**95**	WO2020206044A1	FLEXIBLE TRANSPARENT MEMBRANE LIGHT EMITTING DIODE ARRAY AND SYSTEMS CONTAINING THE SAME	2020-10-08
**96**	CN113950872A	METHOD FOR TRANSFERRING A MATERIAL	2020-10-01
**97**	JP2020160445A	DISPLAY DEVICE AND MANUFACTURING METHOD FOR THE SAME	2020-09-30
**98**	FR3093944A1<br>FR3093944B1	CARTOUCHE POUR BIOIMPRESSION	2020-09-25
**99**	CN114390909A	MANUFACTURING TECHNOLOGY OF SKIN COMPATIBLE ELECTRODE	2020-09-09
**100**	US11185396B2<br>US2020261191A1	3D FABRICATION FOR DENTAL APPLICATIONS BASED ON ABLATION	2020-08-20
**101**	WO2020156632A1<br>WO2020156632A8	METHOD FOR APPLYING AT LEAST ONE SILICON LAYER BY MEANS OF LASER TRANSFER PRINTING	2020-08-06
**102**	WO2020152352A1	LASER INDUCED FORWARD TRANSFER WITH HIGH THROUGHPUT AND RECYCLING OF DONOR MATERIAL ON A TRANSPARENT DRUM	2020-07-30
**103**	CN113227391A	LASER-INDUCED CELL TRANSFER AND SORTING	2020-06-24
**104**	WO2020127391A1	METHOD AND DEVICE FOR PRODUCING SACCHARIDES AND SACCHARIDE ARRAYS	2020-06-24
**105**	CN210826328U	CLAMPING DEVICE FOR LASER-INDUCED FORWARD TRANSFER TECHNOLOGY	2020-06-23
**106**	CN111326548A	DISPLAY DEVICE	2020-06-18
**107**	WO2020123851A2<br>WO2020123851A3	LIGHT EMITTING DIODE (LED) MASS-TRANSFER APPARATUS AND METHOD OF MANUFACTURE	2020-06-18
**108**	EP3663090A1	HIGH RESOLUTION LASER INDUCED FORWARD TRANSFER	2020-06-10
**109**	CN111235545A	NANO-ALLOY PARTICLES AND PATTERNING METHOD THEREOF	2020-06-05
**110**	WO2020108821A1	METHOD FOR LAYER-WISE ADDITIVE MANUFACTURING OF A SHAPED BODY	2020-06-03
**111**	EP3660087A1	METHOD AND MATERIAL FOR THE PRODUCTION OF THREE-DIMENSIONAL OBJECTS BY ENERGY IMPULSE-INDUCED TRANSFER PRINTING	2020-06-03
**112**	EP3660085A1	SUPPORT MATERIAL FOR ENERGY IMPULSE-INDUCED TRANSFER PRINTING	2020-06-03
**113**	EP3659989A1	SLURRY AND METHOD FOR MANUFACTURING OF CERAMIC AND GLASS-CERAMIC 3D STRUCTURES	2020-06-03
**114**	CN113169238A	PHOTOVOLTAIC DEVICE AND METHOD OF MANUFACTURING THE SAME	2020-05-20
**115**	WO2020097083A1	SYSTEMS FOR CELL CONTROL	2020-05-14
**116**	EP3650240A1	MULTIPLE COLOR IMAGE	2020-05-13
**117**	US2021394444A1	ENCLOSED BIOPRINTING DEVICE	2020-04-30
**118**	US2021358792A1	METHOD FOR PRODUCING OPTOELECTRONIC DEVICES	2020-04-30
**119**	CN112930263A	ROBOTIC BIOPRINTING SYSTEM	2020-04-30
**120**	US2021348288A1	MENISCUS-CONFINED THREE-DIMENSIONAL ELECTRODEPOSITION	2020-04-16
**121**	US11213608B2<br>US2020108172A1	COMPOSITIONS INCLUDING GELATIN NANOPARTICLES AND METHODS OF USE THEREOF	2020-04-09
**122**	CN112739953A	A SEGMENTED LIGHT GUIDE AND A METHOD OF MANUFACTURING THEREOF	2020-03-25
**123**	US10600739B1	INTERPOSER WITH INTERCONNECTS AND METHODS OF MANUFACTURING THE SAME	2020-03-24
**124**	EP3624571A1	A PROCESS FOR THE MANUFACTURING OF PRINTED CONDUCTIVE TRACKS ON AN OBJECT AND 3D PRINTED ELECTRONICS	2020-03-18
**125**	KR102146240B1<br>KR20200016099A	METHOD OF FORMING PATTERNS FOR A STRETCHABLE DEVICE	2020-02-14
**126**	CN110756986A	METHOD AND DEVICE FOR PREPARING MICRO LENS ARRAY THROUGH LASER-INDUCED FORWARD TRANSFER	2020-02-07
**127**	WO2020026241A1	TOBACCO TRANSGENIC EVENT AND METHODS FOR DETECTION AND USE THEREOF	2020-02-06
**128**	US2020023584A1	FABRICATION AND DESIGN OF COMPOSITES WITH ARCHITECTED LAYERS	2020-01-23
**129**	CN110690300A<br>CN110690300B	LASER-INDUCED TRANSFER PRINTING METHOD FOR ELECTRODE GRID LINE OF PHOTOVOLTAIC SOLAR CELL	2020-01-14
**130**	CN110666169A<br>CN110666169B	MULTI-MATERIAL LASER-INDUCED FORWARD TRANSFER 3D PRINTING DEVICE AND METHOD	2020-01-10
**131**	FR3081753A1<br>FR3081753B1	SYSTEME DE DEPOT MATIERE ET PROCEDE ASSOCIE	2019-12-05
**132**	WO2019226195A2<br>WO2019226195A3	FABRICATION AND DESIGN OF COMPOSITES WITH ARCHITECTED LAYERS	2019-11-28
**133**	CN110484425A	SINGLE-CELL COLLECTION DEVICE	2019-11-22

**134**	CN209502972U	PROCESSING DEVICE FOR MANUFACTURING SILVER PASTE ELECTRODE THROUGH LASER-INDUCED FORWARD TRANSFER PRINTING AND SINTERING	2019-10-18
**135**	WO2019198086A1	BIOENGINEERED CORNEAL GRAFT AND METHODS OF PREPARATION THEREOF	2019-10-17
**136**	US11453171B2<br>US2021362431A1	METHOD OF APPARATUS FOR FORMING AN OBJECT BY MEANS OF ADDITIVE MANUFACTURING	2019-10-16
**137**	CN111954935A	LASER ASSISTED METALLIZATION PROCESS FOR SOLAR CELL FABRICATION	2019-10-10
**138**	WO2019195793A1	LASER ASSISTED METALLIZATION PROCESS FOR SOLAR CELL STRINGING	2019-10-10
**139**	WO2019195803A1	LASER ASSISTED METALLIZATION PROCESS FOR SOLAR CELL FABRICATION	2019-10-10
**140**	EP3543371A1<br>EP3543371B1	CONTINUOUS LASER INDUCED FORWARD TRANSFER OF MATERIAL SYSTEM	2019-09-25
**141**	US10982109B2<br>US2021047532A1	TWO-COMPONENT PRINTABLE CONDUCTIVE COMPOSITION	2019-09-19
**142**	US2020376486A1	METHODS AND APPARATUS FOR MANUFACTURING A MICROFLUIDIC ARRANGEMENT, AND A MICROFLUIDIC ARRANGEMENT	2019-08-28
**143**	CN209281091U	SOLDER PASTE LASER-INDUCED FORWARD TRANSFER EQUIPMENT	2019-08-20
**144**	WO2019154980A1	METHOD FOR LASER-INDUCED FORWARD TRANSFER USING EFFECT PIGMENTS	2019-08-15
**145**	WO2019154826A1	METHOD FOR LASER-INDUCED FORWARD TRANSFER USING METAL OXIDE ABSORBER PARTICLES	2019-08-15
**146**	CN111684099A	LIFT DEPOSITION APPARATUS AND METHOD	2019-08-07
**147**	WO2019151854A1	METHOD AND APPARATUS FOR CREATING AND SINTERING FINE LINES AND PATTERNS	2019-08-07
**148**	CN111684550A	DIRECT PRINTING OF EMBEDDED RESISTORS	2019-07-18
**149**	WO2019129349A1	DUAL BEAM LASER TRANSFER	2019-07-04
**150**	DE102017130947A1<br>DE102017130947B4	VERFAHREN ZUR HERSTELLUNG EINES MIKROSYSTEMBAUTEILS	2019-06-27
**151**	CN109926583A	PROCESSING DEVICE AND METHOD FOR PREPARING SILVER PASTE ELECTRODE BY LASER-INDUCED FORWARD TRANSFERRING PRINTING AND SINTERING	2019-06-25
**152**	CN109911848A<br>CN109911848B	DEVICE AND METHOD CAPABLE OF PRECISELY CONTROLLING AND TRANSMITTING NANOWIRE	2019-06-21
**153**	US11027145B2<br>US2019184189A1	LASER INDUCED BALLISTIC PARTICLE IMPLANTATION TECHNIQUE	2019-06-20
**154**	CN109877341A<br>CN109877341B	SMELTING METHOD AND PATTERNING METHOD OF NANO METAL PARTICLES	2019-06-14
**155**	EP3710275A1<br>EP3710275B1	METAL DROPLET JETTING SYSTEM	2019-05-16
**156**	US11388824B2<br>US2019110367A1	COMPONENT CARRIER HAVING A THREE DIMENSIONALLY PRINTED WIRING STRUCTURE	2019-04-10
**157**	US10254499B1	ADDITIVE MANUFACTURING OF ACTIVE DEVICES USING DIELECTRIC, CONDUCTIVE AND MAGNETIC MATERIALS	2019-04-09
**158**	CN109581674A<br>CN109581674B	SOLDER PASTE LASER-INDUCED FORWARD TRANSFER DEVICE AND METHOD	2019-04-05
**159**	RO133156A2	METHOD FOR PRINTING PIXELS WITH PIEZOELECTRIC ACTIVITY OF ECOLOGICAL MATERIALS BASED ON DOPED BARIUM TITANATE	2019-03-29
**160**	US2020166438A1	TISSUE MARKING SYSTEM	2019-02-21
**161**	US11155774B2<br>US2018371389A1	METHOD AND APPARATUS FOR GENERATING THREE-DIMENSIONAL PATTERNED SOFT STRUCTURES AND USES THEREOF	2018-12-27
**162**	US2018370116A1	THREE-DIMENSIONAL PRINTING OF REACTIVE MATERIALS USING INTERSECTING JETS	2018-12-27
**163**	WO2018225076A1<br>WO2018225076A8	ADDITIVE MANUFACTURING USING RECOMBINANT COLLAGEN-CONTAINING FORMULATION	2018-12-13
**164**	WO2018225073A1	3D PRINTING OF MEDICINAL UNIT DOSES	2018-12-13
**165**	US2021028141A1	ELECTRICAL INTERCONNECTION OF CIRCUIT ELEMENTS ON A SUBSTRATE WITHOUT PRIOR PATTERNING	2018-11-29
**166**	US10940687B2<br>US2020070514A1	SYSTEM AND METHOD FOR LASER INDUCED FORWARD TRANSFER COMPRISING A MICROFLUIDIC CHIP PRINT HEAD WITH A RENEWABLE INTERMEDIATE LAYER	2018-10-25
**167**	KR20200008558A	ENERGY CONTROL COATINGS, STRUCTURES, DEVICES AND METHODS OF MAKING SAME	2018-10-25
**168**	WO2018193454A1	MICROFLUIDIC HEAD FOR LASER INDUCED FORWARD TRANSFER	2018-10-25
**169**	EP3596197A1<br>EP3596197B1	BIOPRINTING PROCESS	2018-09-20

**170**	CN110402125A<br>CN110402125B	EQUIPMENT AND METHOD FOR ADDITIVE MANUFACTURING	2018-09-20
**171**	WO2018167399A1	DEVICE AND METHOD FOR THE DEPOSITION OF PARTICLES ON A TARGET	2018-09-20
**172**	US10916465B1	INORGANIC LIGHT EMITTING DIODE (ILED) ASSEMBLY VIA DIRECT BONDING	2018-09-13
**173**	WO2018140145A1	COMPOSITIONS AND METHODS RELATED TO 2 DIMENSIONAL MOLECULAR COMPOSITES	2018-08-02
**174**	WO2018127850A1	THREE-DIMENSIONAL TUMOR MODELS, METHODS OF MANUFACTURING SAME AND USES THEREOF	2018-07-12
**175**	JP2020514094A<br>JP6851649B2	METHODS FOR DEPOSITING FUNCTIONAL MATERIALS ON SUBSTRATES	2018-06-28
**176**	US2018171468A1	METHOD FOR DEPOSTING A FUNCTIONAL MATERIAL ON A SUBSTRATE	2018-06-21
**177**	US2019301006A1	COMPOSITION SUITABLE FOR APPLICATION WITH LASER INDUCED FORWARD TRANSFER (LIFT)	2018-06-13
**178**	WO2018100580A1	METHOD AND SYSTEM FOR 3D PRINTING	2018-06-07
**179**	CN109952189A	HYBRID, MULTI-MATERIAL 3D PRINTING	2018-05-24
**180**	CN108018549A	MULTIDIMENSIONAL MICRO STRUCTURE DEPOSITION METHOD BASED ON LASER INDUCED NOZZLE	2018-05-11
**181**	US2021354179A1	SINGLE PROCEDURE INDICATORS	2018-04-26
**182**	WO2018069448A1	FORMING SOLID MATERIAL IN RECESS OF LAYER STRUCTURE BASED ON APPLIED FLUIDIC MEDIUM	2018-04-19
**183**	KR20180035127A	- METHOD AND APPARATUS FOR PRINTING HIGH-VISCOSITY MATERIALS	2018-03-29
**184**	CN109716536A	METHOD FOR PRODUCING ELECTRICAL CONTACTS ON A COMPONENT	2018-03-22
**185**	US2020055327A1	MULTI-TECHNOLOGY PRINTING SYSTEM	2018-02-22
**186**	JP2019532490A	IMPROVED PROCESSING OF POLYMER-BASED INKS AND PASTES	2018-02-14
**187**	EP4086080A1	LASER-INDUCED MATERIAL DISPENSING	2018-01-18
**188**	CN107521239A	MASKLESS PAINTING AND PRINTING	2017-12-20
**189**	CN107378231A<br>CN107378231B	METHOD FOR PREPARING METAL STRUCTURE ON SURFACE OF TRANSPARENT MATERIAL BY USING METAL NANOMETER INK	2017-11-24
**190**	US2017329201A1	TUNABLE ACOUSTIC GRADIENT INDEX OF REFRACTION LENS AND SYSTEM	2017-11-16
**191**	US10957615B2<br>US2019019736A1	LASER-SEEDING FOR ELECTRO-CONDUCTIVE PLATING	2017-10-05
**192**	CA3018405A1	METHOD FOR SOLVENT-FREE PRINTING CONDUCTORS ON SUBSTRATE	2017-09-28
**193**	CN109153037A<br>CN109153037B	METHOD FOR DEPOSITING FUNCTIONAL MATERIAL ON SUBSTRATE	2017-09-21
**194**	US11089690B2<br>US2017268100A1	METHOD FOR DEPOSITING A FUNCTIONAL MATERIAL ON A SUBSTRATE	2017-09-21
**195**	CN206404752U	DEVICE OF PREPARATION PARTICLE	2017-08-15
**196**	GB2546761A<br>GB2546761B	METHODS AND CIRCUITS	2017-08-02
**197**	WO2017103007A1	METHOD FOR ACTIVATING CLICK REACTIONS THROUGH LASER INDUCED FORWARD TRANSFER OF MOLECULES	2017-06-21
**198**	CN106825915A<br>CN106825915B	SYSTEM AND METHOD FOR PREPARING PATTERNED METAL THIN LAYER THROUGH PULSE LASER-INDUCED FORWARD TRANSFER	2017-06-13
**199**	CN106842588A<br>CN106842588B	DEVICE AND METHOD FOR PREPARING STRUCTURAL COLOUR FILM BY LASER-INDUCED FORWARD TRANSFER	2017-06-13
**200**	US10446260B2<br>US2017160535A1	SPATIALLY INDEXED TISSUE BIOBANK WITH MICROSCOPIC PHENOTYPE-BASED RETRIEVAL SYSTEM	2017-06-08
**201**	US10688692B2<br>US2018281243A1	CONTROL OF SURFACE PROPERTIES OF PRINTED THREE-DIMENSIONAL STRUCTURES	2017-05-26
**202**	US10658283B2<br>US2018323139A1	METHOD FOR MANUFACTURING A DEVICE WITH INTEGRATED-CIRCUIT CHIP BY DIRECT DEPOSIT OF CONDUCTIVE MATERIAL	2017-05-10
**203**	US2017103902A1<br>US9685349B2	LASER-INDUCED FORMING AND TRANSFER OF SHAPED METALLIC INTERCONNECTS	2017-04-13
**204**	WO2017044646A1	LASER PROCESSING APPARATUS, METHODS OF LASER-PROCESSING WORKPIECES AND RELATED ARRANGEMENTS	2017-03-16
**205**	KR101787013B1<br>KR20170026718A	APPARATUS FOR FINE PATTERN FORMATION USING ROLL-TO-ROLL PRINTED ELECTRONICS PROCESS	2017-03-09
**206**	US2017028626A1	COMPACT DROP-ON-DEMAND APPARATUS USING LIGHT ACTUATION THROUGH OPTICAL FIBERS	2017-02-02
**207**	WO2017011029A1	STABLE UNDERCOOLED METALLIC PARTICLES FOR ENGINEERING AT AMBIENT CONDITIONS	2017-01-19
**208**	CN107849687A<br>CN107849687B	CONTROL OF LASER-INDUCED FORWARD TRANSFER EJECTION ANGLE	2017-01-12

**209**	KR101694625B1	3D SCAFFOLD FOR TISSUE REGENERATION AND MANUFACTURING METHOD THEREOF	2017-01-09
**210**	WO2017004615A1	ISOLATION OF MICRONICHES FROM SOLID-PHASE AND SOLID SUSPENSION IN LIQUID PHASE MICROBIOMES USING LASER INDUCED FORWARD TRANSFER	2017-01-05
**211**	US2016367358A1<br>US9968437B2	3D PRINTER	2016-12-22
**212**	US2016368211A1	3D PRINTER	2016-12-22
**213**	WO2016189527A1	A TWO, THREE AND FOUR DIMENSIONS BIO PRINTING SYSTEM	2016-12-01
**214**	EP3289838A1<br>EP3289838B1	PRINTING METHOD USING TWO LASERS	2016-10-28
**215**	WO2016124708A1	CONTINUOUS PROCESS FOR OPTIMIZED LASER INDUCED FORWARD TRANSFER TO CREATE ARBITRARY PATTERNS	2016-08-11
**216**	US10633758B2<br>US2017365484A1	PRINTING OF THREE-DIMENSIONAL METAL STRUCTURES WITH A SACRIFICIAL SUPPORT	2016-07-28
**217**	CN107206548A<br>CN107206548B	ANGLED LASER-INDUCED FORWARD CONVERSION JETTING	2016-07-28
**218**	EP3233499A1<br>EP3233499B1	LASER PRINTING METHOD, AND DEVICE FOR IMPLEMENTING SAID METHOD	2016-06-23
**219**	EP3234102A1<br>EP3234102B1	METHOD FOR LASER PRINTING BIOLOGICAL COMPONENTS, AND DEVICE FOR IMPLEMENTING SAID METHOD	2016-06-23
**220**	US10363731B2<br>US2016176110A1	EJECTOR DEVICE	2016-06-23
**221**	EP3035404A1	BARRIER FOIL COMPRISING AN ELECTRICAL CIRCUIT	2016-06-22
**222**	WO2016063270A1	LLIFT PRINTING OF CONDUCTIVE TRACES ONTO A SEMICONDUCTOR SUBSTRATE	2016-04-28
**223**	EP3200974A1<br>EP3200974B1	ALIGNING AN AGENT DISTRIBUTOR	2016-04-07
**224**	US9290671B1	LOW COST SEMICONDUCTING ALLOY NANOPARTICLES INK AND MANUFACTURING PROCESS THEREOF	2016-03-22
**225**	US2017210142A1<br>US9925797B2	LIFT PRINTING SYSTEM	2016-02-11
**226**	EP3172488A1<br>EP3172488B1	LIGHT SOURCE COOLING BODY, LIGHT SOURCE ASSEMBLY, A LUMINAIRE AND METHOD TO MANUFACTURE A LIGHT SOURCE COOLING BODY OR A LIGHT SOURCE ASSEMBLY	2016-01-28
**227**	CN105271106A	LASER IMPLANTATION PREPARATION METHOD FOR MULTI-DIMENSIONAL CONTINUOUS FINE STRUCTURE	2016-01-27
**228**	CN106687617A<br>CN106687617B	LASER-TRANSFERRED IBC SOLAR CELLS	2016-01-21
**229**	WO2015181810A1	PRINTING OF 3D STRUCTURES BY LASER-INDUCED FORWARD TRANSFER	2015-12-03
**230**	WO2015177497A1	ADDITIVE MANUFACTURE OF COMPOSITE MATERIALS	2015-11-26
**231**	GB2526328A	ADDITIVE MANUFACTURE OF COMPOSITE MATERIALS	2015-11-25
**232**	EP2946912A1	ADDITIVE MANUFACTURE OF COMPOSITE MATERIALS	2015-11-25
**233**	US2015301428A1<br>US9594288B2	TUNABLE ACOUSTIC GRADIENT INDEX OF REFRACTION LENS AND SYSTEM	2015-10-22
**234**	TW201537639A<br>TWI640041B	METHODS FOR HIGH-RESOLUTION PATTERNING OF MULTIPLE LAYERS SIDE BY SIDE	2015-10-01
**235**	US2017071062A1<br>US9750141B2	PRINTING HIGH ASPECT RATIO PATTERNS	2015-09-24
**236**	WO2015107051A1	LASER DIRECT SYNTHESIS AND DEPOSIT OF NANOCOMPOSITE MATERIALS OR NANOSTRUCTURES	2015-07-22
**237**	US10820418B2<br>US2016330841A1	ELECTRONIC MODULE, METHOD FOR MANUFACTURING SAME AND ELECTRONIC DEVICE COMPRISING A MODULE OF SAID TYPE	2015-07-08
**238**	JP2020061553A<br>JP7130614B2	PRINTED CIRCUIT WIRING REPAIR	2015-06-24
**239**	EP2883709A1	METHOD AND SYSTEM FOR FORMING A PATTERNED STRUCTURE ON A SUBSTRATE	2015-06-17
**240**	KR102292841B1<br>KR20160078383A	METHOD AND SYSTEM FOR FORMING A PATTERNED STRUCTURE ON A SUBSTRATE	2015-05-07
**241**	WO2015061800A2<br>WO2015061800A3	TISSUE ENGINEERED DEVICES AND METHODS FOR MAKING SAME	2015-04-30
**242**	AU2015101219A4	METHOD FOR PREPARING CELL-LADEN 3D SCAFFOLDS AND APPLICATION	2015-04-29
**243**	TW201525166A<br>TWI623633B	METHOD AND APPARATUS FOR MATERIAL DEPOSITION AND DONOR DEVICE	2015-04-23
**244**	WO2015054188A1	MULTIMODE IMAGE AND SPECTRAL READER	2015-04-16
**245**	WO2015028407A1	METHOD FOR LITHOGRAPHIC PATTERNING OF ORGANIC LAYERS	2015-03-05

**246**	CA2919094A1	INTERFEROMETRIC LASER PROCESSING	2015-01-29
**247**	US10822605B2<br>US2015072873A1	METHOD AND APPARATUS FOR PRODUCING SEQUENCE VERIFIED DNA	2014-11-06
**248**	WO2014172630A1	LASER MARKING FOR AUTHENTICATION AND TRACKING	2014-10-23
**249**	CA2907039A1<br>CA2907039C	ELECTROACTIVE CULTURES AND APPARATUSES THEREFOR	2014-09-25
**250**	CN105143987A<br>CN105143987B	MECHANICALLY PRODUCED ALIGNMENT FIDUCIAL METHOD AND ALIGNMENT SYSTEM	2014-09-18
**251**	CN104995725A	METHOD FOR CREATING ELECTRICAL CONTACTS AND CONTACTS CREATED IN THIS WAY	2014-05-30
**252**	KR20140051042A	MULTI-TECHNOLOGY PRINTING SYSTEM	2014-04-30
**253**	US2017043603A1<br>US9751351B2	MULTI-TECHNOLOGY PRINTING SYSTEM	2014-04-24
**254**	ES2443240A2<br>ES2443240B1<br>ES2443240R1	METHOD FOR THE SELECTIVE DOPING OF A SEMICONDUCTOR BY LASER INDUCED TRANSFER (MACHINE-TRANSLATION BY GOOGLE TRANSLATE, NOT LEGALLY BINDING)	2014-02-18
**255**	EP2685515A1	METHOD AND SYSTEM FOR DIVIDING A BARRIER FOIL	2014-01-15
**256**	EP2660352A1	DONOR SHEET AND METHOD FOR LIGHT INDUCED FORWARD TRANSFER MANUFACTURING	2013-11-06
**257**	EP2844485A1<br>EP2844485B1	DIE FOR DEPOSITING AT LEAST ONE CONDUCTIVE FLUID ONTO A SUBSTRATE, AND DEVICE INCLUDING SUCH A MATRIX AND DEPOSITION METHOD	2013-11-01
**258**	US2014155297A1	METHOD AND APPARATUS FOR LIGHT BASED RECOVERY OF SEQUENCE VERIFIED DNA	2013-08-29
**259**	WO2013124254A1	METHOD FOR CONTACTING A SEMICONDUCTOR SUBSTRATE, MORE PARTICULARLY FOR CONTACTING SOLAR CELLS, AND SOLAR CELLS CONTACTED THEREBY	2013-07-25
**260**	US2013148196A1<br>US9213175B2	MICROSCOPE WITH TUNABLE ACOUSTIC GRADIENT INDEX OF REFRACTION LENS ENABLING MULTIPLE FOCAL PLAN IMAGING	2013-06-13
**261**	US2013141782A1<br>US9256009B2	TUNABLE ACOUSTIC GRADIENT INDEX OF REFRACTION LENS AND SYSTEM	2013-06-06
**262**	WO2013081204A1	LASER PATTERN PROCESSING APPARATUS USING A DIFFRACTIVE OPTIC ELEMENT	2013-06-06
**263**	US2013063805A1	TUNABLE ACOUSTIC GRADIENT INDEX OF REFRACTION LENS AND SYSTEM	2013-03-14
**264**	JP2014522128A<br>JP5753320B2	LITHOGRAPHIC APPARATUS	2013-02-19
**265**	US2013036928A1<br>US9555644B2	NON-CONTACT TRANSFER PRINTING	2013-01-17
**266**	KR20120139892A	METHOD FOR PATTERNING THIN FILM USING VISCOELASTIC FLOW AND SUBSTRATE PATTERNED THE THIN FILM	2012-12-28
**267**	CN103597404A<br>CN103597404B	LITHOGRAPHIC APPARATUS, PROGRAMMABLE PATTERNING DEVICE AND LITHOGRAPHIC METHOD	2012-10-09
**268**	WO2012066338A2<br>WO2012066338A3	METHOD AND APPARATUS FOR DIRECT WRITING	2012-05-24
**269**	KR101116375B1<br>KR20110138447A	METHOD OF MANUFACTURING ADHESIVE METAL FILM AND THE METAL FILM	2011-12-28
**270**	US2013176699A1	METHOD AND APPARATUS FOR DEPOSITION	2011-12-15
**271**	EP2569141A2<br>EP2569141B1	METHOD FOR PRODUCING AN ORGAN REPLICA, IN PARTICULAR A FUNCTIONAL MODEL	2011-11-17
**272**	US2011278566A1	METHOD OF PATTERNING THIN FILM SOLUTION-DEPOSITED	2011-11-17
**273**	JP2013521033A<br>JP5757961B2	BIOPRINTING STATION, ASSEMBLY CONTAINING THE BIOPRINTING STATION, AND BIOPRINTING METHOD	2011-09-09
**274**	US2011240611A1<br>US8743165B2	METHODS AND DEVICE FOR LASER PROCESSING	2011-09-09
**275**	ES2360778A1<br>ES2360778B1	APPARATUS AND METHOD FOR DIRECT LASER PRINTING	2011-01-27
**276**	US2012080088A1	METHOD OF CONTACTING A SEMICONDUCTOR SUBSTRATE	2010-11-11
**277**	KR101039549B1<br>KR20100110447A	METHOD FOR DIRECT-LASER PATTERNING OF THIN FILM	2010-10-13
**278**	US2010227133A1<br>US8663754B2	PULSED LASER MICRO-DEPOSITION PATTERN FORMATION	2010-09-09
**279**	KR101095855B1<br>KR20100051201A	PATTERN CLEANING METHOD AND PATTERN CLEANING DEVICE	2010-05-17
**280**	KR101080672B1<br>KR20100039730A	METHOD AND SYSTEM FOR TRANSFERRING PATTERN	2010-04-16
**281**	KR100973681B1<br>KR20100039734A	SYSTEM AND METHOD FOR TRANSFERRING PATTERN	2010-04-16

**282**	US2010035375A1<br>US7682970B2	MASKLESS NANOFABRICATION OF ELECTRONIC COMPONENTS	2010-02-11
**283**	KR100951778B1<br>KR20090123411A	METHOD FOR PATTERNING THIN FILM BY LASER PRINTING	2009-12-02
**284**	US2009274740A1	DRUG-LOADED MEDICAL DEVICES AND METHODS FOR MANUFACTURING DRUG-LOADED MEDICAL DEVICES	2009-11-05
**285**	US2009217517A1<br>US8056222B2	LASER-BASED TECHNIQUE FOR THE TRANSFER AND EMBEDDING OF ELECTRONIC COMPONENTS AND DEVICES	2009-09-03
**286**	KR100920388B1<br>KR20090079341A	METHOD FOR PATTERNING THIN-FILM BY PHOTORESIST-FREE LITHOGRAPHY	2009-07-22
**287**	TW200945339A	OPTICAL DISK FORMAT FOR DIRECT WRITING MATERIALS ON A SUBSTRATE	2009-07-02
**288**	US2009130427A1	NANOMATERIAL FACILITATED LASER TRANSFER	2009-05-21
**289**	US2011000785A1<br>US8603308B2	ELECTRICAL PATTERNS FOR BIOSENSOR AND METHOD OF MAKING	2009-05-07
**290**	CN101821111A<br>CN101821111B	LASER DECAL TRANSFER OF ELECTRONIC MATERIALS	2009-03-19
**291**	US2008314881A1<br>US8101247B2	SUB-MICRON LASER DIRECT WRITE	2008-12-24
**292**	KR20100016648A	IMPROVED THREE-DIMENSIONAL BIOCOMPATIBLE SKELETON STRUCTURE CONTAINING NANOPARTICLES	2008-10-30
**293**	US2013330850A1<br>US8684779B2	ELECTRODE PATTERNING	2008-10-16
**294**	US2016025903A1	TUNABLE ACOUSTIC GRADIENT INDEX OF REFRACTION LENS AND SYSTEM	2008-09-04
**295**	WO2008047144A1	METHODS AND APPARATUS FOR THE MANUFACTURE OF MICROSTRUCTURES	2008-04-24
**296**	CN101443788A<br>CN101443788B	DEPOSITION REPAIR APPARATUS AND METHODS	2007-11-22
**297**	EP1847632A1<br>EP1847632B1	APPARATUS AND PROCESS FOR THE PREPARATION OF P-TYPE SEMICONDUCTOR ZINC OXIDE FILMS	2007-10-18
**298**	KR100760311B1	PATTERNING METHOD OF DISPLAY DEVICE	2007-09-20
**299**	WO2007071985A1	METHOD OF AND APPARATUS FOR TRANSFERRING MATERIAL FROM A CARRIER TO A SUBSTRATE BY LASER IRRADIATION	2007-06-28
**300**	US2007105395A1	LASER FUNCTIONALIZATION AND PATTERNING OF THICK-FILM INKS	2007-05-10
**301**	WO2006111766A2<br>WO2006111766A3	METHODS AND APPARATUS FOR THE MANUFACTURE OF MICROSTRUCTURES	2006-10-26
**302**	GB2425401A	MANUFACTURE OF MICROSTRUCTURES USING PEELABLE MASK	2006-10-25
**303**	WO2006061658A1	ELECTRODE PATTERNING	2006-06-15
**304**	US7001467B2	DIRECT-WRITE LASER TRANSFER AND PROCESSING	2006-02-21
**305**	US2005288813A1	DIRECT WRITE AND FREEFORM FABRICATION APPARATUS AND METHOD	2005-09-29
**306**	US2005054121A1<br>US7423286B2	LASER TRANSFER ARTICLE AND METHOD OF MAKING	2005-03-10
**307**	US2005015175A1<br>US7277770B2	DIRECT WRITE PROCESS AND APPARATUS	2005-01-20
**308**	KR100543139B1<br>KR20050003723A	CONDUCTIVE PATTERN FABRICATION METHOD FOR ELIMINATING VACUUM PROCESS, MASK, AND PHOTORESIST BY USING DIRECT WIRING METHOD UTILIZING LASER ABLATION	2005-01-12
**309**	KR101314469B1<br>KR20120048038A	BATTERY STRUCTURES, SELF-ORGANIZING STRUCTURES AND RELATED METHODS	2004-06-05
**310**	US2005026037A1<br>US7763382B2	BIPOLAR ARTICLES AND RELATED METHODS	2004-02-05
**311**	WO03101165A1	DIRECT-WRITE LASER TRANSFER AND PROCESSING	2003-12-04
**312**	CN100595964C<br>CN1864298A	BATTERY STRUCTURES, SELF-ORGANIZING STRUCTURES AND RELATED METHODS	2003-02-13
**313**	US2003017277A1<br>US6815015B2	JETTING BEHAVIOR IN THE LASER FORWARD TRANSFER OF RHEOLOGICAL SYSTEMS	2003-01-23
**314**	US2002197401A1<br>US6805918B2	LASER FORWARD TRANSFER OF RHEOLOGICAL SYSTEMS	2002-12-26
**315**	WO02092674A1	LASER FORWARD TRANSFER OF RHEOLOGICAL SYSTEMS	2002-11-21
**316**	WO03056320A2<br>WO03056320A3	FABRICATION OF BIOPOLYMER PATTERNS BY MEANS OF LASER TRANSFER	2002-11-15
**317**	US2002122898A1<br>US6905738B2	GENERATION OF VIABLE CELL ACTIVE BIOMATERIAL PATTERNS BY LASER TRANSFER	2002-09-05
**318**	WO02066550A1	GENERATION OF BIOMATERIAL MICROARRAYS BY LASER TRANSFER	2002-08-29
**319**	US2002071901A1<br>US6936311B2	GENERATION OF BIOMATERIAL MICROARRAYS BY LASER TRANSFER	2002-06-13

**320**	US6177151B1	MATRIX ASSISTED PULSED LASER EVAPORATION DIRECT WRITE	2001-01-23
**321**	WO0044960A1	MATRIX ASSISTED PULSED LASER EVAPORATION DIRECT WRITE	2000-08-03

Patent list from USPTO

#	Number	Title	Publication date
**1**	US 20220347778 A1	LASER PRINTING OF SOLDER PASTES	2022-11-03
**2**	US 20220348859 A1	LITHOGRAPHIC MASKING FOR SPATIALLY LOCALIZED BIOCHEMICAL STIMULUS DELIVERY	2022-11-03
**3**	US 11474290 B2	SEGMENTED LIGHT GUIDE HAVING LIGHT-BLOCKING GROOVE BETWEEN SEGMENTS, AND A METHOD OF MANUFACTURING	2022-10-18
**4**	US 11472373 B2	ENERGY CONTROL COATINGS, STRUCTURES, DEVICES, AND METHODS OF FABRICATION THEREOF	2022-10-18
**5**	US 11476307 B2	PHOTOVOLTAIC DEVICE AND METHOD OF MANUFACTURING THE SAME	2022-10-18
**6**	US 11465173 B2	KIT AND SYSTEM FOR LASER-INDUCED MATERIAL DISPENSING	2022-10-11
**7**	US 11465401 B2	EJECTOR DEVICE	2022-10-11
**8**	US 20220296424 A1	SYSTEM AND METHOD FOR PERSONALIZED IMPLANTABLE SCAFFOLDS FOR WOUND HEALING	2022-09-22
**9**	US 20220293577 A1	DISPLAY DEVICE AND DISPLAY UNIT	2022-09-15
**10**	US 11440241 B2	ADDITIVE MANUFACTURING OF A FREE FORM OBJECT MADE OF MULTICOMPONENT MATERIALS	2022-09-13
**11**	US 20220281253 A1	ADDITIVE OPTO-THERMOMECHANICAL NANOPRINTING AND NANOREPAIRING UNDER AMBIENT CONDITIONS	2022-09-08
**12**	US 20220266382 A1	LASER-SEEDING FOR ELECTRO-CONDUCTIVE PLATING	2022-08-25
**13**	US 20220260063 A1	ELECTROSPRAY EMISSION APPARATUS	2022-08-18
**14**	US 11409184 B2	ACOUSTO-OPTIC DEFLECTOR WITH MULTIPLE OUTPUT BEAMS	2022-08-09
**15**	US 20220248540 A1	HIGH-RESOLUTION SOLDERING	2022-08-04
**16**	US 20220230944 A1	CONFIGURABLE LEADED PACKAGE	2022-07-21
**17**	US 11393773 B1	STRESS ISOLATING INTERPOSER AND SENSOR PACKAGE AND METHOD OF MANUFACTURING THE SAME	2022-07-19
**18**	US 20220219165 A1	METHOD OF MANUFACTURING A MICROFLUIDIC ARRANGEMENT, METHOD OF OPERATING A MICROFLUIDIC ARRANGEMENT, APPARATUS FOR MANUFACTURING A MICROFLUIDIC ARRANGEMENT	2022-07-14
**19**	US 20220208701 A1	PRINTED PACKAGE AND METHOD OF MAKING THE SAME	2022-06-30
**20**	US 20220194111 A1	METHOD AND A DEVICE FOR ASSEMBLY OF A NANOMATERIAL STRUCTURE	2022-06-23
**21**	US 20220187904 A1	FLEXIBLE TRANSPARENT MEMBRANE LIGHT EMITTING DIODE ARRAY AND SYSTEMS CONTAINING THE SAME	2022-06-16
**22**	US 20220192030 A1	CIRCUIT BOARDS HAVING SIDE-MOUNTED COMPONENTS ANS ADDITIVE MANUFACTURINGF METHODS THEREOF	2022-06-16
**23**	US 20220179136 A1	OPTICAL ELEMENT ARRAY STRUCTURE FOR BIRD COLLISION PREVENTION AND MANUFACTURING METHOD THEREOF	2022-06-09
**24**	US 20220167897 A1	MANUFACTURING OF SKIN-COMPATIBLE ELECTRODES	2022-06-02
**25**	US 20220168847 A1	LASER PROCESSING APPARATUS, METHODS OF OPERATING THE SAME, AND METHODS OF PROCESSING WORKPIECES USING THE SAME	2022-06-02
**26**	US 20220160547 A1	DEVICES AND METHODS FOR LASER SURGERY OF AN EYE, ESPECIALLY FOR KERATOPLASTY	2022-05-26
**27**	US 20220152925 A1	THREE-DIMENSIONAL ADDITIVE PRINTING METHOD	2022-05-19
**28**	US 20220088854 A1	METHOD FOR TRANSFERRING A MATERIAL	2022-03-24
**29**	US 20220062849 A1	METHOD AND DEVICE FOR PRODUCING SACCHARIDES AND SACCHARIDE ARRAYS	2022-03-03
**30**	US 20220057721 A1	APPARATUS FOR THE EXPOSURE OF PLATE-SHAPED WORKPIECES WITH HIGH THROUGHPUT	2022-02-24
**31**	US 20220048135 A1	LASER-PROCESSING APPARATUS, METHODS OF OPERATING THE SAME, AND METHODS OF PROCESSING WORKPIECES USING THE SAME	2022-02-17
**32**	US 20220040377 A1	LASER ABLATION/REMOVAL AND LASER INDUCED FORWARD TRANSFER OF BIOLOGICAL MATERIAL	2022-02-10
**33**	US 20220024223 A1	LIFT PRINTING USING THIN DONOR FOILS	2022-01-27
**34**	US 20220016707 A1	METHOD FOR LAYER-WISE MANUFACTURING OF A SHAPED BODY	2022-01-20
**35**	US 20220009247 A1	HIGH RESOLUTION LASER INDUCED FORWARD TRANSFER	2022-01-13
**36**	US 20210408123 A1	PHOTOVOLTAIC DEVICE AND METHOD OF MANUFACTURING THE SAME	2021-12-30

**37**	US 20210404968 A1	INDUCTIVELY COUPLED PLASMA TORCH WITH REVERSE VORTEX FLOW AND METHOD OF OPERATION	2021-12-30
**38**	US 20210403942 A1	SYSTEMS FOR CELL CONTROL	2021-12-30
**39**	US 20210402805 A1	DEVICE	2021-12-30
**40**	US 20210404921 A1	PSORIASIS AND OTHER AUTOIMMUNE DISEASES ANTIGEN IMMUNE MODULATOR (AIM) THERAPEUTIC PLATFORM	2021-12-30
**41**	US 11211569 B2	LASER PRINTABLE ORGANIC SEMICONDUCTOR COMPOSITIONS AND APPLICATIONS THEREOF	2021-12-28
**42**	US 20210394444 A1	ENCLOSED BIOPRINTING DEVICE	2021-12-23
**43**	US 20210379581 A1	MAGNETIC PCR ASSAY AND USES THEREOF	2021-12-09
**44**	US 20210370704 A1	MULTIPLE COLOR IMAGE	2021-12-02
**45**	US 20210373313 A1	AUTOFOCUS SAMPLE IMAGING APPARATUS AND METHOD	2021-12-02
**46**	US 20210370702 A1	LIGHT IRRADIATION METHOD, LIGHT ABSORBING MATERIAL ATTACHING APPARATUS, FLYING BODY GENERATING METHOD AND APPARATUS, IMAGE FORMING METHOD, AND THREE-DIMENSIONAL OBJECT PRODUCING METHOD	2021-12-02
**47**	US 20210362431 A1	METHOD OF APPARATUS FOR FORMING AN OBJECT BY MEANS OF ADDITIVE MANUFACTURING	2021-11-25
**48**	US 20210367140 A1	METHOD FOR APPLYING AT LEAST ONE SILICONE LAYER BY LASER TRANSFER PRINTING	2021-11-25
**49**	US 20210362277 A1	LASER-PROCESSING APPARATUS, METHODS OF OPERATING THE SAME, AND METHODS OF PROCESSING WORKPIECES USING THE SAME	2021-11-25
**50**	US 20210354381 A1	ROBOTIC BIOPRINTING SYSTEM	2021-11-18
**51**	US 20210358792 A1	METHOD FOR PRODUCING OPTOELECTRONIC DEVICES	2021-11-18
**52**	US 20210356376 A1	FUSED-REFERENCE PARTICLE BASED NORMALISATION FOR IMAGING MASS SPECTROMETRY	2021-11-18
**53**	US 20210348288 A1	MENISCUS-CONFINED THREE-DIMENSIONAL ELECTRODEPOSITION	2021-11-11
**54**	US 20210346985 A1	FRAME AND EXTERIOR SHROUDING FOR LASER PROCESSING SYSTEM	2021-11-11
**55**	US 20210339539 A1	LASER-INDUCED CELL TRANSFER AND SORTING	2021-11-04
**56**	US 20210339469 A1	APPARATUS CONFIGURED TO MODEL THREE-DIMENSIONAL MODELED OBJECT, APPARATUS CONFIGURED TO FLY PARTICLES, AND METHOD OF MODELING THREE-DIMENSIONAL MODELED OBJECT	2021-11-04
**57**	US 20210333173 A1	HIGH SPEED MODULATION SAMPLE IMAGING APPARATUS AND METHOD	2021-10-28
**58**	US 20210310082 A1	TOBACCO TRANSGENIC EVENT AND METHODS FOR DETECTION AND USE THEREOF	2021-10-07
**59**	US 20210286120 A1	A SEGMENTED LIGHT GUIDE AND A METHOD OF MANUFACTURING THEREOF	2021-09-16
**60**	US 11117217 B1	METAL FILM FOR ADDITIVE METAL MANUFACTURING	2021-09-14
**61**	US 20210282269 A1	PRINTED CIRCUIT BOARDS IMPREGNATED WITH CARBON NANO TUBES	2021-09-09
**62**	US 20210252782 A1	THREE-DIMENSIONAL MODELING APPARATUS AND THREE-DIMENSIONAL MODELING METHOD	2021-08-19
**63**	US 20210239707 A1	REAGENTS AND METHODS FOR ELEMENTAL MASS SPECTROMETRY OF BIOLOGICAL SAMPLES	2021-08-05
**64**	US 11077664 B2	SYSTEMS AND METHODS FOR CONTROLLING THE MORPHOLOGY AND POROSITY OF PRINTED REACTIVE INKS FOR HIGH PRECISION PRINTING	2021-08-03
**65**	US 20210229983 A1	METHOD FOR CLOSING OPENINGS IN A FLEXIBLE DIAPHRAGM OF A MEMS ELEMENT	2021-07-29
**66**	US 20210227697 A1	A PROCESS FOR THE MANUFACTURING OF PRINTED CONDUCTIVE TRACKS ON AN OBJECT AND 3D PRINTED ELECTRONICS	2021-07-22
**67**	US 20210205813 A1	CONTACTLESS LIQUID LOADING TO MICROFLUIDIC DEVICES	2021-07-08
**68**	US 11053156 B2	METHOD OF CLOSED FORM RELEASE FOR BRITTLE MATERIALS USING BURST ULTRAFAST LASER PULSES	2021-07-06
**69**	US 20210181186 A1	REAGENTS AND METHODS FOR ELEMENTAL IMAGING MASS SPECTROMETRY OF BIOLOGICAL SAMPLES	2021-06-17
**70**	US 20210168943 A1	METHOD FOR MANUFACTURING A CONDUCTOR STRUCTURAL ELEMENT AND CONDUCTOR STRUCTURAL ELEMENT	2021-06-03
**71**	US 20210155023 A1	ARCHITECTED STAMPS FOR LIQUID TRANSFER PRINTING	2021-05-27
**72**	US 20210157238 A1	METHODS AND SYSTEMS FOR PRODUCING THREE-DIMENSIONAL ELECTRONIC PRODUCTS	2021-05-27
**73**	US 20210139321 A1	LASER-ASSISTED MATERIAL PHASE-CHANGE AND EXPULSION MICRO-MACHINING PROCESS	2021-05-13
**74**	US 20210129433 A1	SYSTEMS FOR MATERIAL DEPOSITION	2021-05-06
**75**	US 20210118661 A1	HIGH RESOLUTION IMAGING APPARATUS AND METHOD	2021-04-22
**76**	US 20210107091 A1	METHOD AND SYSTEM FOR EXTENDING OPTICS LIFETIME IN LASER PROCESSING APPARATUS	2021-04-15
**77**	US 20210094125 A1	METHOD FOR MICROWELDING FLEXIBLE THIN FILMS, FOR EXAMPLE FOR USE IN ELECTRICAL AND ELECTRONIC DEVICES	2021-04-01

**78**	US 20210087670 A1	LIFT DEPOSITION APPARATUS AND METHOD	2021-03-25
**79**	US 20210090945 A1	SYSTEM AND METHOD FOR INTERCONNECTION	2021-03-25
**80**	US 20210069971 A1	SYSTEM FOR DEPOSITING MATERIAL AND ASSOCIATED METHOD	2021-03-11
**81**	US 20210053297 A1	METHODS AND SYSTEMS FOR CONTROLLING TEMPERATURE ACROSS A REGION DEFINED BY USING THERMALLY CONDUCTIVE ELEMENTS	2021-02-25
**82**	US 20210057311 A1	METHOD AND SYSTEM OF STRETCHING AN ACCEPTOR SUBSTRATE TO ADJUST PLACEMENT OF A COMPONENT	2021-02-25
**83**	US 20210047532 A1	TWO-COMPONENT PRINTABLE CONDUCTIVE COMPOSITION	2021-02-18
**84**	US 20210045252 A1	SYSTEMS AND METHODS FOR MANUFACTURING	2021-02-11
**85**	US 20210032739 A1	DYNAMIC RELEASE MIRROR STRUCTURE FOR LASER-INDUCED FORWARD TRANSFER	2021-02-04
**86**	US 20210023772 A1	METHOD AND APPARATUS FOR CREATING AND SINTERING FINE LINES AND PATTERNS	2021-01-28
**87**	US 20210028141 A1	ELECTRICAL INTERCONNECTION OF CIRCUIT ELEMENTS ON A SUBSTRATE WITHOUT PRIOR PATTERNING	2021-01-28
**88**	US 10883136 B2	METHOD OF ISOLATING BIOCHEMICAL MOLECULES ON MICROARRAY SUBSTRATE	2021-01-05
**89**	US 20200387009 A1	CONTACT LENSES WITH MICROCHANNELS	2020-12-10
**90**	US 20200376486 A1	METHODS AND APPARATUS FOR MANUFACTURING A MICROFLUIDIC ARRANGEMENT, AND A MICROFLUIDIC ARRANGEMENT	2020-12-03
**91**	US 20200371107 A1	METHOD FOR SELECTING, MANIPULATING AND ISOLATING CIRCULATING TUMOR CELLS IN BODY FLUIDS BY LASER-ASSISTED TRANSFER	2020-11-26
**92**	US 20200354660 A1	SYSTEM AND METHOD FOR ORGANOID CULTURE	2020-11-12
**93**	US 20200350275 A1	METHOD TO ELECTRICALLY CONNECT CHIP WITH TOP CONNECTORS USING 3D PRINTING	2020-11-05
**94**	US 10808794 B1	TOPOLOGICAL DAMPING MATERIALS AND METHODS THEREOF	2020-10-20
**95**	US 20200324564 A1	DUAL BEAM LASER TRANSFER	2020-10-15
**96**	US 20200261191 A1	3D FABRICATION FOR DENTAL APPLICATIONS BASED ON ABLATION	2020-08-20
**97**	US 20200250856 A1	CALIBRATION OF A LIGHT-FIELD IMAGING SYSTEM	2020-08-06
**98**	US 20200215633 A1	SOLDER PASTE LASER INDUCED FORWARD TRANSFER DEVICE AND METHOD	2020-07-09
**99**	US 20200194721 A1	DISPLAY DEVICE	2020-06-18
**100**	US 20200194616 A1	LIGHT EMITTING DIODE (LED) MASS-TRANSFER APPARATUS AND METHOD OF MANUFACTURE	2020-06-18
**101**	US 20200179562 A1	ADDITIVE MANUFACTURING USING RECOMBINANT COLLAGEN-CONTAINING FORMULATION	2020-06-11
**102**	US 20200172444 A1	SLIP AND PROCESS FOR THE PRODUCTION OF CERAMIC AND GLASS CERAMIC 3D STRUCTURES	2020-06-04
**103**	US 20200171700 A1	SUPPORT MATERIAL FOR ENERGY-PULSE-INDUCED TRANSFER PRINTING	2020-06-04
**104**	US 20200172747 A1	PROCESS AND MATERIAL FOR PRODUCING 3D OBJECTS BY ENERGY-PULSE-INDUCED TRANSFER PRINTING	2020-06-04
**105**	US 20200166438 A1	TISSUE MARKING SYSTEM	2020-05-28
**106**	US 20200139935 A1	ENERGY CONTROL COATINGS, STRUCTURES, DEVICES, AND METHODS OF FABRICATION THEREOF	2020-05-07
**107**	US 20200108172 A1	COMPOSITIONS INCLUDING GELATIN NANOPARTICLES AND METHODS OF USE THEREOF	2020-04-09
**108**	US 20200102529 A1	EQUIPMENT AND METHOD FOR ADDITIVE MANUFACTURING	2020-04-02
**109**	US 20200096448 A1	METHOD AND DEVICE FOR PROVIDING A CELL LINE HAVING A DESIRED TARGET PROTEIN EXPRESSION	2020-03-26
**110**	US 10600739 B1	INTERPOSER WITH INTERCONNECTS AND METHODS OF MANUFACTURING THE SAME	2020-03-24
**111**	US 20200070514 A1	SYSTEM AND METHOD FOR LASER INDUCED FORWARD TRANSFER COMPRISING A MICROFLUIDIC CHIP PRINT HEAD WITH A RENEWABLE INTERMEDIATE LAYER	2020-03-05
**112**	US 20200046520 A1	BIOPRINTING PROCESS	2020-02-13
**113**	US 20200033359 A1	IMMUNE PROFILING OF TUMOR TISSUE	2020-01-30
**114**	US 20200023584 A1	FABRICATION AND DESIGN OF COMPOSITES WITH ARCHITECTED LAYERS	2020-01-23
**115**	US 20200009877 A1	DEVICE AND METHOD FOR THE DEPOSITION OF PARTICLES ON A TARGET	2020-01-09
**116**	US 20190367884 A1	THREE-DIMENSIONAL TUMOR MODELS, METHODS OF MANUFACTURING SAME AND USES THEREOF	2019-12-05
**117**	US 20190352430 A1	COMPOSITIONS AND METHODS RELATED TO 2 DIMENSIONAL MOLECULAR COMPOSITES	2019-11-21
**118**	US 10451953 B2	ACOUSTO-OPTIC DEFLECTOR WITH MULTIPLE OUTPUT BEAMS	2019-10-22

**119**	US 10446720 B2	SEMICONDUCTOR STRUCTURE, LIGHT-EMITTING DEVICE AND MANUFACTURING METHOD FOR THE SAME	2019-10-15
**120**	US 20190312157 A1	LASER ASSISTED METALLIZATION PROCESS FOR SOLAR CELL FABRICATION	2019-10-10
**121**	US 20190312163 A1	LASER ASSISTED METALLIZATION PROCESS FOR SOLAR CELL STRINGING	2019-10-10
**122**	US 20190308270 A1	SYSTEMS FOR LASER ASSISTED METALLIZATION OF SUBSTRATES	2019-10-10
**123**	US 20190301006 A1	COMPOSITION SUITABLE FOR APPLICATION WITH LASER INDUCED FORWARD TRANSFER (LIFT)	2019-10-03
**124**	US 20190279771 A1	SRM/MRM ASSAYS FOR PROFILING TUMOR TISSUE	2019-09-12
**125**	US 20190263054 A1	HYBRID, MULTI-MATERIAL 3D PRINTING	2019-08-29
**126**	US 10391588 B2	METHOD AND SYSTEM FOR SCRIBING BRITTLE MATERIAL FOLLOWED BY CHEMICAL ETCHING	2019-08-27
**127**	US 20190237599 A1	METHOD FOR PRODUCING ELECTRICAL CONTACTS ON A COMPONENT	2019-08-01
**128**	US 20190193444 A1	METHOD AND SYSTEM FOR APPLYING A PATTERNED STRUCTURE ON A SURFACE	2019-06-27
**129**	US 20190184189 A1	LASER INDUCED BALLISTIC PARTICLE IMPLANTATION TECHNIQUE	2019-06-20
**130**	US 20190169457 A1	PROCESSING OF POLYMER BASED INKS AND PASTES	2019-06-06
**131**	US 20190143449 A1	METAL DROPLET JETTING SYSTEM	2019-05-16
**132**	US 20190110367 A1	COMPONENT CARRIER HAVING A THREE DIMENSIONALLY PRINTED WIRING STRUCTURE	2019-04-11
**133**	US 10252507 B2	METHOD AND APPARATUS FOR FORWARD DEPOSITION OF MATERIAL ONTO A SUBSTRATE USING BURST ULTRAFAST LASER PULSE ENERGY	2019-04-09
**134**	US 10254499 B1	ADDITIVE MANUFACTURING OF ACTIVE DEVICES USING DIELECTRIC, CONDUCTIVE AND MAGNETIC MATERIALS	2019-04-09
**135**	US 20190104618 A1	METHOD FOR SOLVENT-FREE PRINTING CONDUCTORS ON SUBSTRATE	2019-04-04
**136**	US 20190094113 A1	CELL SORTING	2019-03-28
**137**	US 10214833 B1	ADDITIVE MANUFACTURING OF CRYSTALLINE MATERIALS	2019-02-26
**138**	US 10191550 B1	FABRIC DEVICES WITH SHAPE MEMORY ALLOY WIRES THAT PROVIDE HAPTIC FEEDBACK	2019-01-29
**139**	US 20190019736 A1	LASER-SEEDING FOR ELECTRO-CONDUCTIVE PLATING	2019-01-17
**140**	US 20190001442 A1	LASER PROCESSING APPARATUS, METHODS OF LASER-PROCESSING WORKPIECES AND RELATED ARRANGEMENTS	2019-01-03
**141**	US 20190001434 A1	LOCATION OF IMAGE PLANE IN A LASER PROCESSING SYSTEM	2019-01-03
**142**	US 20180370116 A1	THREE-DIMENSIONAL PRINTING OF REACTIVE MATERIALS USING INTERSECTING JETS	2018-12-27
**143**	US 20180371389 A1	METHOD AND APPARATUS FOR GENERATING THREE-DIMENSIONAL PATTERNED SOFT STRUCTURES AND USES THEREOF	2018-12-27
**144**	US 20180354074 A1	METHOD FOR JOINING TWO COMPONENTS IN THE REGION OF A JOINT ZONE BY MEANS OF AT LEAST ONE LASER BEAM, AND METHOD FOR GENERATING A CONTINUOUS JOINT SEAM	2018-12-13
**145**	US 10144088 B2	METHOD AND APPARATUS FOR LASER PROCESSING OF SILICON BY FILAMENTATION OF BURST ULTRAFAST LASER PULSES	2018-12-04
**146**	US 20180323139 A1	METHOD FOR MANUFACTURING A DEVICE WITH INTEGRATED-CIRCUIT CHIP BY DIRECT DEPOSIT OF CONDUCTIVE MATERIAL	2018-11-08
**147**	US 20180281243 A1	CONTROL OF SURFACE PROPERTIES OF PRINTED THREE-DIMENSIONAL STRUCTURES	2018-10-04
**148**	US 20180261582 A1	INORGANIC LIGHT EMITTING DIODE (ILED) ASSEMBLY VIA DIRECT BONDING	2018-09-13
**149**	US 20180193948 A1	CONTROL OF LIFT EJECTION ANGLE	2018-07-12
**150**	US 10005152 B2	METHOD AND APPARATUS FOR SPIRAL CUTTING A GLASS TUBE USING FILAMENTATION BY BURST ULTRAFAST LASER PULSES	2018-06-26
**151**	US 20180171468 A1	METHOD FOR DEPOSTING A FUNCTIONAL MATERIAL ON A SUBSTRATE	2018-06-21
**152**	US 20180112277 A1	SIGNAL DIRECTED DISSECTION TO INFORM CANCER THERAPY STRATEGY	2018-04-26
**153**	US 20180111177 A1	SINGLE PROCEDURE INDICATORS	2018-04-26
**154**	US 20180110127 A1	PRINTING METHOD USING TWO LASERS	2018-04-19
**155**	US 20180090314 A1	METHODS AND APPARATUS FOR PRINTING HIGH-VISCOSITY MATERIALS	2018-03-29
**156**	US 9925509 B2	METHOD FOR COMBINATORIAL PARTICLE MANIPULATION FOR PRODUCING HIGH-DENSITY MOLECULE ARRAYS, IN PARTICULAR PEPTIDE ARRAYS, AND MOLECULE ARRAYS THAT CAN BE OBTAINED BY MEANS THEREOF	2018-03-27
**157**	US 20180074457 A1	NEAR-TO-EYE AND SEE-THROUGH HOLOGRAPHIC DISPLAYS	2018-03-15
**158**	US 20180050550 A1	MULTI-TECHNOLOGY PRINTING SYSTEM	2018-02-22
**159**	US 9878536 B2	ACOUSTOPHORETIC PRINTING APPARATUS AND METHOD	2018-01-30
**160**	US 20180015671 A1	RECURRING PROCESS FOR LASER INDUCED FORWARD TRANSFER AND HIGH THROUGHPUT AND RECYCLING OF DONOR MATERIAL BY THE	2018-01-18

		REUSE OF A PLURALITY OF TARGET SUBSTRATE PLATES OR FORWARD TRANSFER OF A PATTERN OF DISCRETE DONOR DOTS	
**161**	US 20180015502 A1	KIT AND SYSTEM FOR LASER-INDUCED MATERIAL DISPENSING	2018-01-18
**162**	US 20170368822 A1	LASER PRINTING METHOD AND DEVICE FOR IMPLEMENTING SAID METHOD	2017-12-28
**163**	US 20170361346 A1	MASKLESS PAINTING AND PRINTING	2017-12-21
**164**	US 20170365484 A1	PRINTING OF THREE-DIMENSIONAL METAL STRUCTURES WITH A SACRIFICIAL SUPPORT	2017-12-21
**165**	US 20170320263 A1	METHOD FOR LASER PRINTING BIOLOGICAL COMPONENTS, AND DEVICE FOR IMPLEMENTING SAID METHOD	2017-11-09
**166**	US 20170306495 A1	ANGLED LIFT JETTING	2017-10-26
**167**	US 20170268100 A1	METHOD FOR DEPOSITING A FUNCTIONAL MATERIAL ON A SUBSTRATE	2017-09-21
**168**	US 9765934 B2	THERMALLY MANAGED LED ARRAYS ASSEMBLED BY PRINTING	2017-09-19
**169**	US 9757815 B2	METHOD AND APPARATUS FOR PERFORMING LASER CURVED FILAMENTATION WITHIN TRANSPARENT MATERIALS	2017-09-12
**170**	US 20170250294 A1	LIFT PRINTING OF CONDUCTIVE TRACES ONTO A SEMICONDUCTOR SUBSTRATE	2017-08-31
**171**	US 20170210142 A1	LIFT PRINTING SYSTEM	2017-07-27
**172**	US 20170205063 A1	LIGHT SOURCE COOLING BODY, LIGHT SOURCE ASSEMBLY, A LUMINAIRE AND METHOD TO MANUFACTURE A LIGHT SOURCE COOLING OR A LIGHT SOURCE ASSEMBLY	2017-07-20
**173**	US 20170189995 A1	PRINTING OF 3D STRUCTURES BY LASER-INDUCED FORWARD TRANSFER	2017-07-06
**174**	US 20170161428 A1	SPATIAL GENOMICS WITH CO-REGISTERED HISTOLOGY	2017-06-08
**175**	US 20170136694 A1	ADDITIVE MANUFACTURE OF COMPOSITE MATERIALS	2017-05-18
**176**	US 20170103902 A1	LASER-INDUCED FORMING AND TRANSFER OF SHAPED METALLIC INTERCONNECTS	2017-04-13
**177**	US 20170072504 A1	QUBITS BY SELECTIVE LASER-MODULATED DEPOSITION	2017-03-16
**178**	US 20170071062 A1	PRINTING HIGH ASPECT RATIO PATTERNS	2017-03-09
**179**	US 20170028626 A1	COMPACT DROP-ON-DEMAND APPARATUS USING LIGHT ACTUATION THROUGH OPTICAL FIBERS	2017-02-02
**180**	US 20170014958 A1	STABLE UNDERCOOLED METALLIC PARTICLES FOR ENGINEERING AT AMBIENT CONDITIONS	2017-01-19
**181**	US 20170002344 A1	ISOLATION OF MICRONICHES FROM SOLID-PHASE AND SOLID SUSPENSION IN LIQUID PHASE MICROBIOMES USING LASER INDUCED FORWARD TRANSFER	2017-01-05
**182**	US 20160367358 A1	3D PRINTER	2016-12-22
**183**	US 20160368211 A1	3D PRINTER	2016-12-22
**184**	US 9517929 B2	METHOD OF FABRICATING ELECTROMECHANICAL MICROCHIPS WITH A BURST ULTRAFAST LASER PULSES	2016-12-13
**185**	US 20160330841 A1	ELECTRONIC MODULE, METHOD FOR MANUFACTURING SAME AND ELECTRONIC DEVICE COMPRISING A MODULE OF SAID TYPE	2016-11-10
**186**	US 20160259250 A1	METHOD AND SYSTEM FOR FORMING A PATTERNED STRUCTURE ON A SUBSTRATE	2016-09-08
**187**	US 20160246892 A1	MULTIMODE IMAGE AND SPECTRAL READER	2016-08-25
**188**	US 20160243286 A1	TISSUE ENGINEERED DEVICES AND METHODS FOR MAKING SAME	2016-08-25
**189**	US 20160233089 A1	LIFT PRINTING OF MULTI-COMPOSITION MATERIAL STRUCTURES	2016-08-11
**190**	US 20160194590 A1	STRUCTURED BIOLOGICAL SAMPLES FOR ANALYSIS BY MASS CYTOMETRY	2016-07-07
**191**	US 20160176110 A1	EJECTOR DEVICE	2016-06-23
**192**	US 20160172595 A1	METHOD FOR LITHOGRAPHIC PATTERNING OF ORGANIC LAYERS	2016-06-16
**193**	US 20160158886 A1	INTERFEROMETRIC LASER PROCESSING	2016-06-09
**194**	US 9328366 B2	METHOD FOR MASS PRODUCTION OF HIGH-PURITY OLIGONUCLEOTIDES	2016-05-03
**195**	US 9290671 B1	LOW COST SEMICONDUCTING ALLOY NANOPARTICLES INK AND MANUFACTURING PROCESS THEREOF	2016-03-22
**196**	US 20160076088 A1	LASER MARKING FOR AUTHENTICATION AND TRACKING	2016-03-17
**197**	US 9284409 B2	ENDOPROSTHESIS HAVING A NON-FOULING SURFACE	2016-03-15
**198**	US 20160038647 A1	THIN-FILM COATED FLUOROPOLYMER CATHETER	2016-02-11
**199**	US 20160036083 A1	ELECTROACTIVE CULTURES AND APPARATUSES THEREFOR	2016-02-04
**200**	US 20160020343 A1	LASER-TRANSFERRED IBC SOLAR CELLS	2016-01-21
**201**	US 20150374920 A1	METHOD AND DEVICE FOR INJECTING A DRUG	2015-12-31
**202**	US 9222753 B2	RETICLE FOR A TELESCOPE	2015-12-29
**203**	US 20150294872 A1	METHOD FOR CREATING ELECTRICAL CONTACTS AND CONTACTS CREATED IN THIS WAY	2015-10-15
**204**	US 20150239274 A1	MULTI-TECHNOLOGY PRINTING SYSTEM	2015-08-27

**205**	US 20150200370 A1	METHOD AND SYSTEM FOR DIVIDING A BARRIER FOIL	2015-07-16
**206**	US 20150122174 A1	DIE FOR DEPOSITING AT LEAST ONE CONDUCTIVE FLUID ONTO A SUBSTRATE, AND DEVICE INCLUDING SUCH A MATRIX AND DEPOSITION METHOD	2015-05-07
**207**	US 20150086705 A1	DONOR SHEET AND METHOD FOR LIGHT INDUCED FORWARD TRANSFER MANUFACTURING	2015-03-26
**208**	US 20150072873 A1	METHOD AND APPARATUS FOR PRODUCING SEQUENCE VERIFIED DNA	2015-03-12
**209**	US 8932346 B2	MEDICAL DEVICES HAVING INORGANIC PARTICLE LAYERS	2015-01-13
**210**	US 8920491 B2	MEDICAL DEVICES HAVING A COATING OF INORGANIC MATERIAL	2014-12-30
**211**	US 8916796 B2	METHOD FOR DEPOSITING AND CURING NANOPARTICLE-BASED INK	2014-12-23
**212**	US 8900292 B2	COATING FOR MEDICAL DEVICE HAVING INCREASED SURFACE AREA	2014-12-02
**213**	US 20140308669 A1	METHODS FOR OBTAINING SINGLE CELLS AND APPLICATIONS OF SINGLE CELL OMICS	2014-10-16
**214**	US 20140287953 A1	LASER ABLATION CELL	2014-09-25
**215**	US 20140268088 A1	MECHANICALLY PRODUCED ALIGNMENT FIDUCIAL METHOD AND DEVICE	2014-09-18
**216**	US 20140238592 A1	SELECTIVE LASER-ASSISTED TRANSFER OF DISCRETE COMPONENTS	2014-08-28
**217**	US 8815275 B2	COATINGS FOR MEDICAL DEVICES COMPRISING A THERAPEUTIC AGENT AND A METALLIC MATERIAL	2014-08-26
**218**	US 8815273 B2	DRUG ELUTING MEDICAL DEVICES HAVING POROUS LAYERS	2014-08-26
**219**	US 8771343 B2	MEDICAL DEVICES WITH SELECTIVE TITANIUM OXIDE COATINGS	2014-07-08
**220**	US 8753855 B2	METHOD FOR DETACHING CULTURED CELLS, CELL DETACHMENT DEVICE USED IN SAID METHOD FOR DETACHING CULTURED CELLS, AND INCUBATOR	2014-06-17
**221**	US 20140160452 A1	LITHOGRAPHIC APPARATUS, PROGRAMMABLE PATTERNING DEVICE AND LITHOGRAPHIC METHOD	2014-06-12
**222**	US 20140155297 A1	METHOD AND APPARATUS FOR LIGHT BASED RECOVERY OF SEQUENCE VERIFIED DNA	2014-06-05
**223**	US 8734915 B2	FILM-FORMATION METHOD AND MANUFACTURING METHOD OF LIGHT-EMITTING DEVICE	2014-05-27
**224**	US 20140071421 A1	LITHOGRAPHIC APPARATUS, PROGRAMMABLE PATTERNING DEVICE AND LITHOGRAPHIC METHOD	2014-03-13
**225**	US 20130335504 A1	OPTICAL WRITER FOR FLEXIBLE FOILS	2013-12-19
**226**	US 8574615 B2	MEDICAL DEVICES HAVING NANOPOROUS COATINGS FOR CONTROLLED THERAPEUTIC AGENT DELIVERY	2013-11-05
**227**	US 20130176699 A1	METHOD AND APPARATUS FOR DEPOSITION	2013-07-11
**228**	US 8449603 B2	ENDOPROSTHESIS COATING	2013-05-28
**229**	US 20130130306 A1	ZN (II) BASED COLORIMETRIC SENSOR AND PROCESS FOR THE PREPARATION THEREOF	2013-05-23
**230**	US 8431149 B2	COATED MEDICAL DEVICES FOR ABLUMINAL DRUG DELIVERY	2013-04-30
**231**	US 20130059280 A1	METHOD FOR PRODUCING AN ORGAN REPLICA, IN PARTICULAR A FUNCTIONAL MODEL	2013-03-07
**232**	US 20130036928 A1	NON-CONTACT TRANSFER PRINTING	2013-02-14
**233**	US 20130017564 A1	BIOPRINTING STATION, ASSEMBLY COMPRISING SUCH BIOPRINTING STATION AND BIOPRINTING METHOD	2013-01-17
**234**	US 8353949 B2	MEDICAL DEVICES WITH DRUG-ELUTING COATING	2013-01-15
**235**	US 8287937 B2	ENDOPROSTHESE	2012-10-16
**236**	US 8231980 B2	MEDICAL IMPLANTS INCLUDING IRIDIUM OXIDE	2012-07-31
**237**	US 8221822 B2	MEDICAL DEVICE COATING BY LASER CLADDING	2012-07-17
**238**	US 8216632 B2	ENDOPROSTHESIS COATING	2012-07-10
**239**	US 8187620 B2	MEDICAL DEVICES COMPRISING A POROUS METAL OXIDE OR METAL MATERIAL AND A POLYMER COATING FOR DELIVERING THERAPEUTIC AGENTS	2012-05-29
**240**	US 20120080088 A1	METHOD OF CONTACTING A SEMICONDUCTOR SUBSTRATE	2012-04-05
**241**	US 8070797 B2	MEDICAL DEVICE WITH A POROUS SURFACE FOR DELIVERY OF A THERAPEUTIC AGENT	2011-12-06
**242**	US 8071156 B2	ENDOPROSTHESES	2011-12-06
**243**	US 8067054 B2	STENTS WITH CERAMIC DRUG RESERVOIR LAYER AND METHODS OF MAKING AND USING THE SAME	2011-11-29
**244**	US 8066763 B2	DRUG-RELEASING STENT WITH CERAMIC-CONTAINING LAYER	2011-11-29
**245**	US 20110278269 A1	METHOD AND SYSTEM FOR ELECTRICAL CIRCUIT REPAIR	2011-11-17
**246**	US 20110278566 A1	METHOD OF PATTERNING THIN FILM SOLUTION-DEPOSITED	2011-11-17
**247**	US 20110240611 A1	METHODS AND DEVICE FOR LASER PROCESSING	2011-10-06
**248**	US 8029554 B2	STENT WITH EMBEDDED MATERIAL	2011-10-04
**249**	US 20110207328 A1	METHODS AND APPARATUS FOR THE MANUFACTURE OF MICROSTRUCTURES	2011-08-25
**250**	US 8002823 B2	ENDOPROSTHESIS COATING	2011-08-23

**251**	US 7981150 B2	ENDOPROSTHESIS WITH COATINGS	2011-07-19
**252**	US 7976915 B2	ENDOPROSTHESIS WITH SELECT CERAMIC MORPHOLOGY	2011-07-12
**253**	US 7942926 B2	ENDOPROSTHESIS COATING	2011-05-17
**254**	US 7938855 B2	DEFORMABLE UNDERLAYER FOR STENT	2011-05-10
**255**	US 7931683 B2	ARTICLES HAVING CERAMIC COATED SURFACES	2011-04-26
**256**	US 7927454 B2	METHOD OF PATTERNING A SUBSTRATE	2011-04-19
**257**	US 20110028344 A1	BIOMARKERS FOR ENDOMETRIAL DISEASE	2011-02-03
**258**	US 20110000785 A1	ELECTRICAL PATTERNS FOR BIOSENSOR AND METHOD OF MAKING	2011-01-06
**259**	US 20100227133 A1	PULSED LASER MICRO-DEPOSITION PATTERN FORMATION	2010-09-09
**260**	US 20100177519 A1	ELECTRO-HYDRODYNAMIC GAS FLOW LED COOLING SYSTEM	2010-07-15
**261**	US 20100120145 A1	THREE-DIMENSIONAL BIOCOMPATIBLE SKELETON STRUCTURE CONTAINING NANOPARTICLES	2010-05-13
**262**	US 20100035375 A1	MASKLESS NANOFABRICATION OF ELECTRONIC COMPONENTS	2010-02-11
**263**	US 20090274740 A1	DRUG-LOADED MEDICAL DEVICES AND METHODS FOR MANUFACTURING DRUG-LOADED MEDICAL DEVICES	2009-11-05
**264**	US 20090217517 A1	LASER-BASED TECHNIQUE FOR THE TRANSFER AND EMBEDDING OF ELECTRONIC COMPONENTS AND DEVICES	2009-09-03
**265**	US 20090130427 A1	NANOMATERIAL FACILITATED LASER TRANSFER	2009-05-21
**266**	US 20090074987 A1	LASER DECAL TRANSFER OF ELECTRONIC MATERIALS	2009-03-19
**267**	US 20080314881 A1	SUB-MICRON LASER DIRECT WRITE	2008-12-25
**268**	US 20080310055 A1	REPEATABLE SHORTING AND UNSHORTING OF MICRO-ELECTRICAL CIRCUITS	2008-12-18
**269**	US 20080252210 A1	ELECTRODE PATTERNING	2008-10-16
**270**	US 20080139075 A1	DEPOSITION REPAIR APPARATUS AND METHODS	2008-06-12
**271**	US 20070243328 A1	P-TYPE SEMICONDUCTOR ZINC OXIDE FILMS PROCESS FOR PREPARATION THEREOF, AND PULSED LASER DEPOSITION METHOD USING TRANSPARENT SUBSTRATES	2007-10-18
**272**	US 20070180991 A1	METHODS FOR PROVIDING THIN HYDROGEN SEPERATION MEMBRANES AND ASSOCIATED USES	2007-08-09
**273**	US 20070105395 A1	LASER FUNCTIONALIZATION AND PATTERNING OF THICK-FILM INKS	2007-05-10
**274**	US 20060234163 A1	LASER-ASSISTED DEPOSITION	2006-10-19
**275**	US 20060213886 A1	MARKING METHOD AND MARKET OBJECT	2006-09-28
**276**	US 20060169441 A1	ELECTRO-HYDRODYNAMIC GAS FLOW COOLING SYSTEM	2006-08-03
**277**	US 20060044702 A1	REPEATABLE ESD PROTECTION	2006-03-02
**278**	US 7001467 B2	DIRECT-WRITE LASER TRANSFER AND PROCESSING	2006-02-21
**279**	US 20050288813 A1	DIRECT WRITE AND FREEFORM FABRICATION APPARATUS AND METHOD	2005-12-29
**280**	US 20050054121 A1	LASER TRANSFER ARTICLE AND METHOD OF MAKING	2005-03-10
**281**	US 6862490 B1	DLL CIRCUIT TAKING ACOUNT OF EXTERNAL LOAD	2005-03-01
**282**	US 20050026037 A1	BIPOLAR ARTICLES AND RELATED METHODS	2005-02-03
**283**	US 20050015175 A1	DIRECT WRITE PROCESS AND APPARATUS	2005-01-20
**284**	US 6792326 B1	MATERIAL DELIVERY SYSTEM FOR MINIATURE STRUCTURE FABRICATION	2004-09-14
**285**	US 6766764 B1	MATRIX ASSISTED PULSED LASER EVAPORATION DIRECT WRITE	2004-07-27
**286**	US 20030178395 A1	METHOD AND APPARATUS FOR FABRICATION OF MINIATURE STRUCTURES	2003-09-25
**287**	US 20030157271 A1	METHOD AND APPARATUS FOR PULSE-POSITION SYNCHRONIZATION IN MINIATURE STRUCTURES MANUFACTURING PROCESSES	2003-08-21
**288**	US 6583381 B1	APPARATUS FOR FABRICATION OF MINIATURE STRUCTURES	2003-06-24
**289**	US 20030099884 A1	BATTERY STRUCTURES, SELF-ORGANIZING STRUCTURES AND RELATED METHODS	2003-05-29
**290**	US 20030017277 A1	JETTING BEHAVIOR IN THE LASER FORWARD TRANSFER OF RHEOLOGICAL SYSTEMS	2003-01-23
**291**	US 20020197401 A1	LASER FORWARD TRANSFER OF RHEOLOGICAL SYSTEMS	2002-12-26
**292**	US 20020122898 A1	GENERATION OF VIABLE CELL ACTIVE BIOMATERIAL PATTERNS BY LASER TRANSFER	2002-09-05
**293**	US 6440503 B1	LASER DEPOSITION OF ELEMENTS ONTO MEDICAL DEVICES	2002-08-27
**294**	US 20020071901 A1	GENERATION OF BIOMATERIAL MICROARRAYS BY LASER TRANSFER	2002-06-13
**295**	US 20020029956 A1	METHOD AND APPARATUS FOR REMOVING MINUTE PARTICLES FROM A SURFACE	2002-03-14
**296**	US 6248658 B1	METHOD OF FORMING SUBMICRON-DIMENSIONED METAL PATTERNS	2001-06-19
**297**	US 6177151 B1	MATRIX ASSISTED PULSED LASER EVAPORATION DIRECT WRITE	2001-01-23
**298**	US 6159832 A	PRECISION LASER METALLIZATION	2000-12-12
**299**	US 6025110 A	METHOD AND APPARATUS FOR GENERATING THREE-DIMENSIONAL OBJECTS USING ABLATION TRANSFER	2000-02-15
**300**	US 5492861 A	PROCESS FOR APPLYING STRUCTURED LAYERS USING LASER TRANSFER	1996-02-20

**301**	US 5281575 A	LASER ABLATION METHOD FOR FORMING OXIDE SUPERCONDUCTING FILMS	1994-01-25
**302**	US 4987006 A	LASER TRANSFER DEPOSITION	1991-01-22
**303**	US 4970196 A	METHOD AND APPARATUS FOR THE THIN FILM DEPOSITION OF MATERIALS WITH A HIGH POWER PULSED LASER	1990-11-13
